# Hierarchical chromatin features reveal the toxin production in *Bungarus multicinctus*

**DOI:** 10.1186/s13020-021-00502-6

**Published:** 2021-09-17

**Authors:** Xuejiao Liao, Shuai Guo, Xianmei Yin, Baosheng Liao, Mingqian Li, He Su, Qiushi Li, Jin Pei, Jihai Gao, Juan Lei, Xiwen Li, Zhihai Huang, Jiang Xu, Shilin Chen

**Affiliations:** 1 Pharmacy College, Chengdu University of Traditional Chinese Medicine, Chengdu, 611137 China; 2 Institute of Chinese Materia Medica, China Academy of Chinese Medical Sciences, Beijing, 100700 China; 3 Guangdong Provincial Hospital of Chinese Medicine, Guangzhou, 510006 China; 4 College of Life Sciences, Beijing Normal University, Beijing, 100875 China

**Keywords:** Hi-C, Chromatin interaction, Chromosome organization, Hierarchical architecture, *Bungarus multicinctus*

## Abstract

**Background:**

*Bungarus multicinctus*, from which a classical Chinese medicine is produced, is known as the most venomous land snake in the world, but the chromatin organization and transcription factor activity during venom replenishment progress have not been explored yet. This study aimed to determine the roles of chromatin structure in toxin activity via bioinformatics and experimental validation.

**Methods:**

Chromosome conformation capture (Hi-C) analysis was used to examine interactions among chromosomes and identify different scales of chromatin during envenomation in *B. multicinctus*. Correlations between epigenetic modifications and chromatin structure were verified through ChIP-seq analysis. RNA-seq was used to validate the influence of variation in chromatin structure and gene expression levels on venom production and regulation.

**Results:**

Our results suggested that intra-chromosomal interactions are more intense than inter-chromosomal interactions among the control group, 3-day group of venom glands and muscles. Through this, we found that compartmental transition was correlated with chromatin interactions. Interestingly, the up-regulated genes in more compartmental switch regions reflect the function of toxin activity. Topologically associated domain (TAD) boundaries enriched with histone modifications are associated with different distributions of genes and the expression levels. Toxin-coding genes in the same loop are highly expressed, implying that the importance of epigenetic regulation during envenomination. On a smaller scale, the epigenetic markers affect transcriptional regulation by controlling the recruitment/inhibition of transcription initiation complexes.

**Conclusions:**

Chromatin structure and epigenetic modifications could play a vital status role in the mechanisms of venom regulation in *B. multicinctus*.

**Supplementary Information:**

The online version contains supplementary material available at 10.1186/s13020-021-00502-6.

## Background

China has a long history of using snakes, dating back to the Spring and Autumn Period and the Warring States Period. The Compendium of Materia Medica written by Li Shizhen in the Ming Dynasty also contains abundant knowledge on the medicinal use of snakes. Snakes are mainly used for medicine, food and tanning, among which there are as many as 70 species of snakes for medicine. The dried body of young *B. multicinctus*, has a remarkable effect on the treating of syphilis and moss scabies. *B. multicinctus* is among the most venomous land snake species in the world according to its median lethal dose (0.09–0.108 mg/kg). The main components of the venom gland system of *B. multicinctus* are proteins, polypeptides, enzymes and other small molecules [1]. Some proteins can also play variety pharmacological actions while producing toxic effects, and snake venom drugs have been proven to treat human diseases [2–5].

3D architecture, including packing chromatin status, conformation and the interactions among inter- or intra-chromosomes plays an important role in regulating the expression of genes [6–8]. Recent work elucidated epigenetic changes that occurred during the development of mammals [9]. Moreover, the correspondence between different regulating elements can be reflected at the gene expression level by epigenetic marks [6, 10, 11]. However, the relationship between the change in the status of chromatin and venom production in *B. multicinctus* is still poorly understood.

In the nucleus of eukaryotes, highly folded chromatin has tight hierarchical structures [12]. Based on the microscopic resolution, the analysis of chromatin can reveal only a rough and general architecture of the nuclei. However, a genome-wide chromatin interaction map has been depicted because of the discovery of 3C-based genome architectures, such as Hi-C [13]. An advanced Hi-C approach targeting different spatial conformations and structural units of chromatin in the nucleus and exploring the interactions between genes and transcriptional regulatory elements regulated by various kinds of structural units has approved the mechanism of gene function and transcriptional regulation [14–16].

Several studies have shown that chromosomes are organized into chromosomal territories during interphase [13, 17]. Moreover, chromosomal territories partition into numerous domains that fall into A/B compartments [18]. On the same chromosome, the frequency of intra-compartmental interactions is much more intense than inter-compartmental interactions. Among them, A compartments are associated with high gene density, higher active transcriptions and epigenetic marks, moreover B compartments are associated with lower gene density, repressive transcription and epigenetic marks [19–21].

At the sub-megabase scale, compartments consist of TADs with 100 kb to 1 Mb scale [9, 22]. As 3D genome units, TADs are considered as the cluster of interactions with many genes in epigenetic states [16, 23–26]. Moreover, architectural proteins such as CCCTC-binding factors (CTCF) and cohesin play an essential role in the formation of TADs. The inter-TAD interactions are linked to cohesin and spatial isolation depends on CTCF. Moreover, intra-TAD interaction frequency is much more than the frequency inter-TAD interactions and the interactions in A/B compartments [9]. TADs have been proven to be evolutionarily conserved in various cell types or even in different species with co-regulated gene clusters [9, 22]. Increasing evidence also highlights the significance of TADs, as functional units during transcription, can regulate the growth and development of mammals by regulating the transcription factor-associated interaction level [9, 27–29]. However, the relationship between transcription and TADs in reptiles is still unknown.

The concentration and aggregation of chromatin is present in the nucleus of eukaryotes; chromatin loops strongly interact between pairs of genomic sites with long ranges in the linear genome [15, 30, 31]. It has been proven that the ends of the loops are usually associated with known gene promoters and enhancers, and these loop-related promoters have higher gene expression levels and greater cell specificity because the function of regulating gene expression [32–34].

Here, we performed a gene-wide chromosome conformation capture method to characterize chromatin interactions in different tissues and times during envenomation in *B. multicinctus*. Our results revealed the features of the composition and fundamental structure of nuclear chromatin in *B. multicinctus* and the connection between epigenetic modifications and the expression of venom-related gene families. This work also provided an essential foundation of the composition and synthesis mechanisms in venom, promoting the development of effective snake venom antiserum.

## Materials and methods

### Ethical approvals

All snakes were obtained from the Julong Artificial Breeding and Farming Center for Special Economic Animals, Liu an, Anhui Province, China. The experiments protocols were approved by the Ethics Committee of the Institute of Chinese Materia Medica, China Academy of Chinese Medical Sciences (approval number ICMM 17–08).

### Reference genome and annotation

Hi-C and ChIP-seq data are available from the National Center for Biotechnology Information (https://www.ncbi.nlm.nih.gov/) Short Read Archive (SRA) under accession number: PRJNA682532. The Illumina RNA-seq data are available at NCBI under BioProject number: PRJNA606820.

### Preparation of Hi-C libraries

Hi-C might have been performed as formerly depicted for minor adjustments. Part of the planned protocol change involved the biotin dosing process, in which the mixture was incubated at 37 °C for 40 min, continuously shaken every ten minutes. The time- and tissue-dependent Hi-C samples showed high efficiency of biotin incorporation of 40% to 85%. After the preparation of the Hi-C samples, a Hi-Seq 2500 instrument was used to sequence the libraries by PE100 reads.

### Analysis of Hi-C data

Raw reads were trimmed Skewer v0.22. Juicer Tools v1.14 was used for Hi-C analysis [35]. Specifically, the contact matrix was extracted using a dump, and data from different tissues were normalized to 1*10^9^ input read pairs before comparison. To find out the differences between time- and tissue-dependent interactions, we calculated the interactions in the Hi-C matrix with 18 chromosomes. We also calculated the length of 18 chromosomes and assigned 11 macro-chromosomes (MACs)(> 50 Mb) and 7 micro-chromosomes (MICs)(< 50 Mb) and distinguish differences in interaction between MACs and MICs.

When developing the principal component analysis method [13, 20], the first principal component generally examines the way in which all genes interact with each other to increase and decrease, which occurs in a grid pattern in the heat map. The genomic regions tend to be matched by highlighting the interaction pattern (values ​​of the positive eigenvector) or vice versa (values ​​of the negative eigenvector), which means two areas are separated spatially. However, in all known analysis, a compartment is likely to end with a positive or negative eigenvector. We calculated the genomic gene density with a bin size of 100 kb by eigenvector to assign the A/B compartment, which then identified the identify A compartment and B compartment.

TADs were calculated using an arrowhead with a bin size of 10 kb. The mean value of the interactions between each bin was computed. Bedtools was used to identify the time- and tissue-specific TADs [36]. With Hi-C interactions, we used matrix2insulation.pl to obtain TAD boundaries. On account of the boundary variations between replicates, we chose to add a total of 80 kb (40 kb of each side) in the boundary to account for replicate variation. And 90-kb regions were defined as the final TAD boundaries in *B. multicinctus*. Similarly, for all boundaries at different times and in various tissues, we detected overlapping boundaries with high reproducibility between biological replicates (~ 80%, ~ 83%, and ~ 93%TAD boundary overlaps for muscle, the control and 3d group of venom gland, respectively). Juicer HICCUPS was used to call loops out with 10 kb and 25 kb lengths and the maximum genomic distance of 1 Mb.

### ChIP and data analysis

ChIP-seq was generally followed V.G. Tim et al. [37]. One percent formaldehyde-fixed muscle and venom gland were used for ChIP-seq, and each tissue was analyzed in triplicates. Antibodies ab47915 (abcam) for H3ac and #9733 (CST) for H3K27me3 were used for immunoprecipitation. The sensitivity and specificity were detected by Western-blot. Then, the precipitated DNA was enriched, purified and fragmented for subsequent sequencing. Illumina TrueSeq Sample Prep Kit was used to construct libraries. All libraries were sequenced by Hiseq X Ten.

Raw reads were trimmed by Skewer v0.22. Bwa v0.7.17 (mem -t 120 -k 18), and Samtools v1.9.0 (view -F 4 -q 30 -bS -@ 8) was used to map and filter reads. Peak calling was performed by MACS2 v2.2.7. Chipseeker v1.22.1 was used for downstream annotations [38], and Homer package v3.2.1 was used for motif finding [39].

### RNA-seq and gene expression analysis

Total RNA was extracted from heart, lung, muscle, kidney, liver, and venom gland using Qiagen RNeasy Kits, each for biological triplicates. The RNA-seq libraries and RNA-seq were generated with Illumina mRNA-seq Prep Kits and Illumina Hiseq X Ten instrument, respectively. Venom replenishment data were obtained from earlier work, the data of which were deposited at NCBI PRJNA608620. Raw reads were trimmed and filtered using Skewer v0.22 [40]. HISAT v2.1.0[41] was used to map trimmed reads to the *B. multicinctus* genome. StringTie v2.0.0 [42] was used to calculate transcript generation and counts. Read count and FPKM values were subjected to further analysis. The R package DEseq2 v1.26.0 was used to calculate differentially expressed gene expression [43].

## Results

### High-resolution Hi-C maps of chromatin interactions in *B. multicinctus*

To obtain the genome-wide chromatin structure of different tissues in physiological states in *B. multicinctus*, we calculated the expression differences with a checkpoint every three days (Control, 0d, 3d, 6d, 9d) after venom expulsion. Generally, variations among replenishment stages were far less than variations among tissues (Kolmogorov–Smirnov test, p = 5.16e-4). For the replenishment process, the gene expression dramatically changed between 3d and the control group point with 277 genes up-regulated and 324 genes down-regulated (log_2_ fold change, lfc > 1, Padj < 0.01) (Additional file 1: Table S1). As for tissues (heart, kidney, lung, liver, muscle, venom gland), it has shown by PCA decomposition of certain principal components that muscle had the most significant differences from the venom glands (Additional file 6: Fig. S1).

After that we performed the Hi-C experiment on the venom gland (control,3d) and muscle. A high-quality Hi-C sequencing library constructed by the MboI enzyme generated 81.7, 55.6 and 102.8 million clean paired end (PE) reads, and 66.65%,68.64% and 19.45% reads were uniquely mapped to the venom gland (control, 3d) and muscle in the *B. multicinctus* reference genome, respectively (Additional file 2: Table S2). After filtration, accurately mapped reads were used to construct an initial interaction matrix. Next, we used the Iterative Correction and Eigenvector Decomposition (ICE) method and the expected/observed normalization, ultimately generating a 2-dimensional Hi-C interaction map of 18 chromosomes in *B. multicinctus* [44]. According to the Hi-C ligation, assembled sequences were assigned to 11 MACs(> 50 Mb) and 7 MICs(< 50 Mb) with N50 of 149.80 Mb, which are under reported cytogenetic karyotyping (Additional file 7: Fig. S2, S3, S4) [45].

**Fig. 1:**
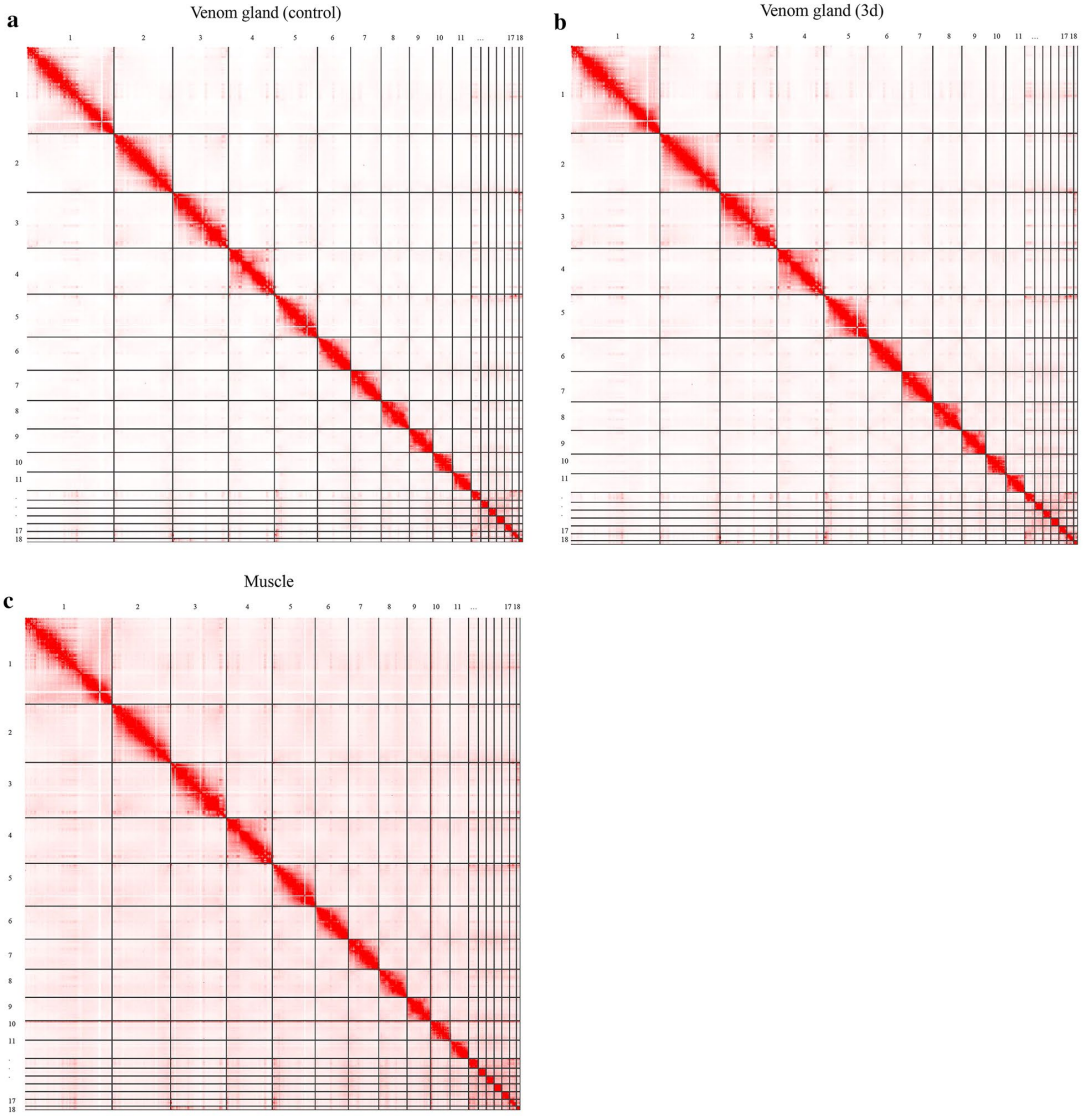
Hi-C contact map. Hi-C interactions within and among *Bungarus multicinctus* chromosomes (Chr1–Chr18). The red color with intensity indicates the frequency of contact. Intra-chromosomal interactions were observed much more frequency than inter-chromosomal interactions. The light-colored rows and columns indicate the bins with no valid interactions

Hi-C heat map revealed two aspects of genome organization (time and tissue) in *B. multicinctus* and indicated strong interactions on the main diagonal, which meant frequent interactions between adjacent loci (Fig. 1). Interestingly, we observed that more intense intra-chromosomal interactions than inter-chromosomal interactions (t-test, P < 0.05) among 18 chromosomes in both venom gland of control, 3d and muscle group (Additional file 7: Figs. S2–S4). On the other hand, the data were in tune with the chromosome territories concept that each chromosome had its own limited nuclear subspace [46], which can facilitate the intra-chromosomal interactions. Interestingly, we further found out that small and gene-enriched chromosomes have more interactions in both the venom gland and muscle. The frequency of inter-chromosomal interactions between inter-MICs through Chr12-Chr18 was significantly larger than the number for junctions of inter/intra-MACs, intra-MICs and junctions across MACs/MICs. The results demonstrated that compared with other chromosomes, the chromosomal territories occupied by MICs were much closer spatially. We compared genome-wide interaction differences to assess whether the gene cluster was altered among control venom gland, 3d venom gland and muscle. Through the analysis, approximately 9.73% of read-pairs were detected as Hi-C contacts in the muscle dataset; for venom glands of control and 3d groups, approximately 50.46% and 50.79%, respectively, of read-pairs were observed (Additional file 2: Table S2).

### The interaction frequency in venom gland is related to the transformation of compartment

Previous research has shown that there are two kinds of interaction patterns have been defined in the genome. A compartment is enriched for open chromatin, and the B compartment is highly enriched for the closed chromatin [13]. For *B. multicinctus* between venom gland (control,3d) and muscle, the interaction maps can also observe two compartmentalization patterns with alternating positive and negative eigenvectors.

**Fig. 2:**
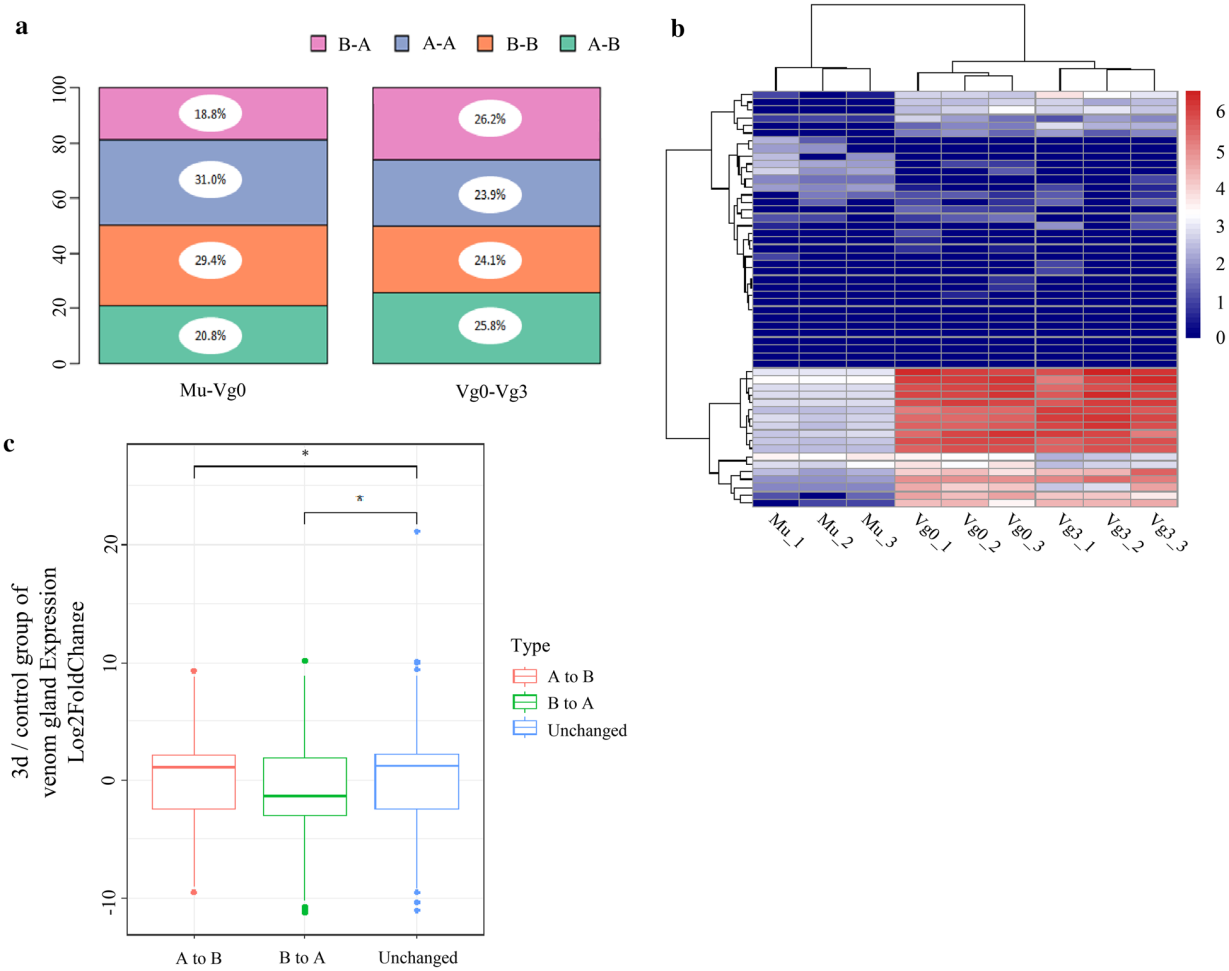
DEGs are enriched in tissue- and time-specific genomic compartments. **a** Histogram showing that the compartmental transformation between different tissues and times. **b** Heatmap shows the main toxin-coding genes in the muscle (Mu), control (Vg0) and 3d group (Vg3) of venom gland among the biological replicates. The scale on the left indicates the expression level on three different groups. **c** Boxplot of log2 fold change expression in 3d/control group of venom gland against the genes locating regions for various compartmental switches. The compartmental switches that are from A to B and B to A indicate significant differences from the unchanged group. Wilcoxon rank-sum test was used to calculate p-value

In an attempt to determine the differences between tissues and times in *B. multicinctus* genomes, we used 100 kb resolution to compare A/B compartments. Most composition of compartments were alike among different tissues (muscle and control group of venom gland) and times (control and 3d group of venom gland). Compartment switching was homogenous through 18 chromosomes, where 31.0 and 29.4% of compartments constituted of A compartment and B compartment in the muscle and control groups of the venom gland, respectively; meanwhile 23.9 and 24.1% made up the A compartment and B compartment in the control and 3d venom gland, respectively (Fig. 2a).

Moreover, approximately 39.6% of the compartment transformed to the opposite compartment (A compartment to B compartment or B compartment to A compartment) in different tissues (venom gland and muscle); 52.0% of the compartments transitioned to the opposite compartment (A compartment to B compartment or B compartment to A compartment) at different times (control and 3d group of venom gland) (Fig. 2a). At the chromosomal level, about 24.9% of all compartments of Chr1, 4 and 5 in the muscle and Chr1, 2 and 4 in the venom gland three days after envenomation changed from B compartment into A compartment versus the control group of venom gland. Chr2 was the chromosome that enriched with the phospholipases A2 (PLA2) family. A recent study proved that there was a close relationship between (small) inter-chromosomal interaction changes and compartmental transformation (Additional file 8: Fig. S5) [47]. We tried to determine whether the compartmental transformation was related to interactions in small chromosomes. We found that not only in different tissues but also at various replenishment times, the increase or decrease in compartmental transition were alike among small chromosomes. However, for Chr12, 14 and16 in various tissues and Chr12, 13, 15 and 16 at different times, about 10% of their total length was converted from A compartment into B compartment. Moreover, the transition to the opposite compartment was significant in the small compartment. According to the results, we suggest that there may be a relationship between chromosomal compartmentalization and gene expression.

### The conversion of A/B compartment is associated with the expression of toxin-coding genes and heterochromatic epigenetic markers

We speculated that there was a correlation between compartmental transformation and venom gland specific gene expression. First, we calculated the differences in gene expression in various tissues and times. For the replenishment process, the gene expression analysis identified that there were 277 up-regulated and 324 down-regulated genes, followed by venom gland versus muscle point with 1669 genes up-regulated and 1684 genes down-regulated (lfc > 1, Padj < 0.01) (Additional file 1: Table S1). As earlier study said that during the different stage of venomous evolution on *B. multicinctus*, PLA2, three-finger toxins(3FTx) and Kunitz-type inhibitors are vital protein families. Thus, we focused on those three kinds of toxin-coding genes, compared with muscle group we found that both in control and 3d group of venom gland had significant highly expressed genes (Fig. 2b). And the Gene Ontology terms associated with toxin-coding genes included terms such as " toxin activity," " metal ion binding " and " serine-type endopeptidase inhibitor activity. "

To determine whether compartmental (open or close) transition was related to the up-regulated or down-regulated genes, we analyzed the significantly expressed gene regions where the compartmental transition occurred more frequently during different times in venom gland. We hypothesized that the significantly expressed genes were enriched in the regions that had more compartmental switch (Fig. 2c). Genes in these regions were shared with the venom gland specific expressed genes, including gene families such as "toxin activity," "protein folding," and " metalloaminopeptidase activity " (Additional file 3: Tables S3, S4).

Finally, to assess whether the epigenetic modifications play a vital role in chromatin architecture at the compartmental level, we also detected histone modifications, including the active marker H3ac and the commonly suppressing marker H3K27me3 for both venom gland and muscle. We found that active histone modifications (H3ac) were significantly enriched in A compartment, whereas suppressing histone modifications (H3K27me3) were significantly enriched in B compartment. The same situation was identified in the chromosomes which enriched with toxin-coding genes. Moreover, H3ac was positively correlated with gene expressions (R = 0.56), and H3K27me3 negatively correlated with the gene expression (R = -0.45) (Additional file 9: Figure S6). In the venom gland, H3ac was enriched in gene families such as "protein folding," "intracellular protein transport," "chromatin silencing" and H3K27me3 were enriched in "mRNA splicing," "translation," "rRNA processing following the chromatin organization features."

### A/B compartments are composed of TAD-interior regions and heterogeneous TAD-boundary

**Fig. 3:**
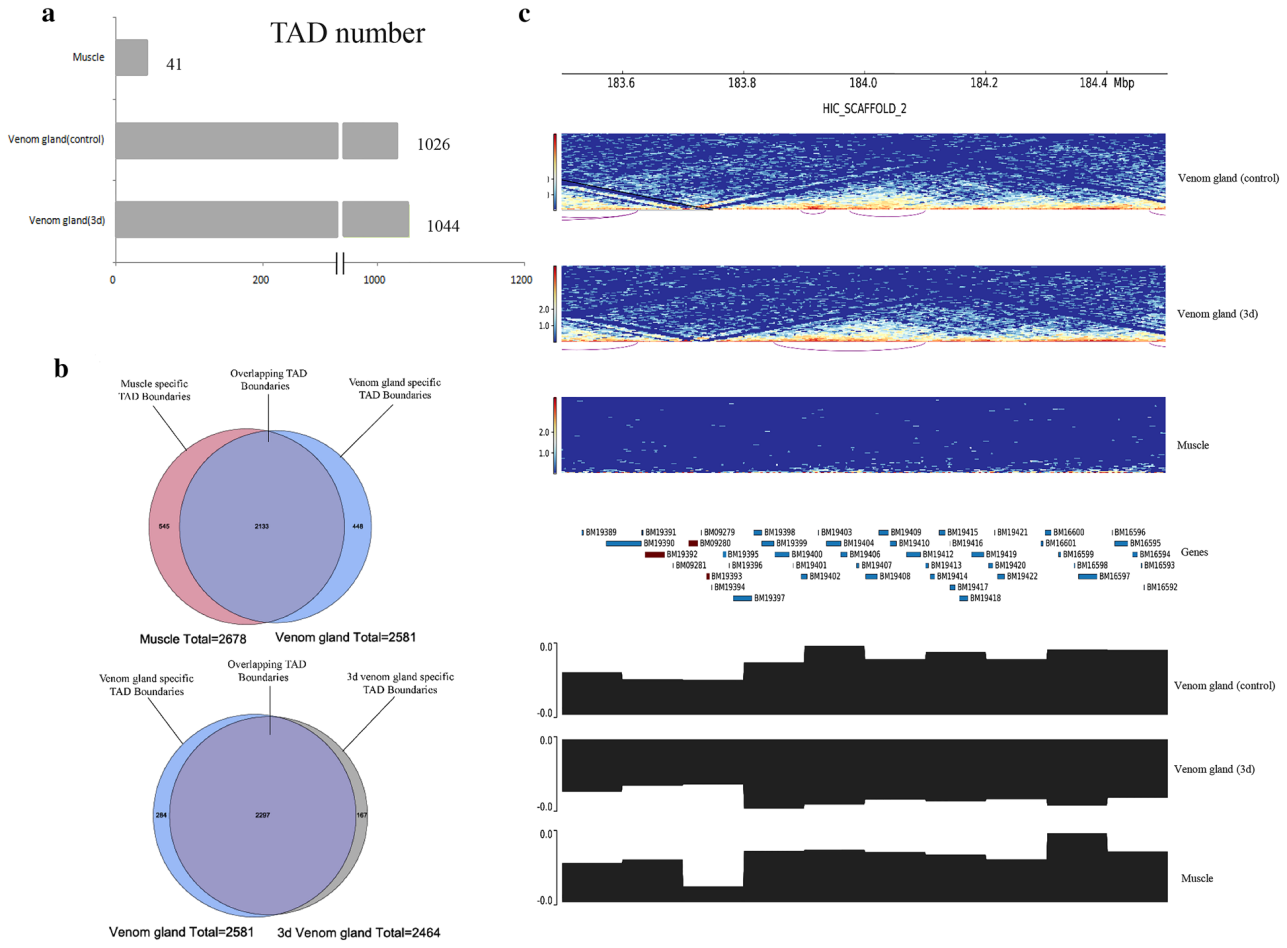
TADs are stable in both time- and tissue- dependent groups. **a** The number of TADs among the control group of venom gland, 3d venom gland and muscle, respectively. **b** Venn diagram shows that the most (83% and 93%) TAD boundaries between the muscle/venom gland and control/3d group of venom gland are conserved. **c** The TAD regions in Chr2: 183 500 000–184 500 000: from upper to the bottom panels, which illustrate the TADs interactions in the control and 3d group of venom gland and muscle group, genes in the 1 M regions of TAD, the compartmental transition from A to B and B to A

At the medium distance scale, A/B compartments consist of TADs with 100 kb to 1 Mb scale, and in a single TAD the level of gene expression can be co-regulated [9, 22, 48]. One specific feature of the mammalian TADs is that they are proved to be stable in various species and cell types [9, 22]. After that, we studied the features of TADs in reptiles. Whether the interaction in chromosomes and the transition of the genomic compartment between time- and tissue-dependent groups have an influence on the structure of TADs and finally on gene expression is still unknown. First, by the use of the Hi-C matrix, 1026, 1044 and 41 TADs were identified to the control of venom gland, 3d venom gland and muscle, respectively (Additional file 4: Table S5, Fig. 3a). All TADs in muscle can be found in venom glands, implying that TADs were conserved among different tissues; in contrast, the remaining TADs in venom gland were preferred to be tissue-specific. For the time-dependent group, 49 and 78 specific TADs were detected in the control and 3d of venom gland, respectively (Additional file 4: Table S6).

In additional to the TADs, we also analyzed the TAD boundaries. In order to know the differences in TAD boundaries, we calculated TAD boundaries by genome-wide interaction maps at the insulation plot of 10 kb resolution. Although there were numerous differences in chromosomal structure, compartmental conversion and gene expression, approximately 80%, 83% and 93% of the TAD boundaries were common among muscle, the control and 3d group of venom gland, respectively (Fig. 3b). Then we analyzed whether tissue- and time-specific TADs had differential gene expression, and the results indicated that the specific type of the TADs was not directly related to DEGs. In addition, we counted the number of DEGs in TADs and TAD boundaries among chromosomes and found that there were obviously more in TAD boundaries than in TADs. Interestingly, there was a boundary aggregation on Chr2, and 303, 285 and 314 TAD boundaries were identified, and among them, three toxin-coding genes (Group I PLA2) were found in venom gland (Fig. 3c, Additional file 4: Table S7).

TAD boundaries were enriched for several classical factors. Classical boundary elements can stop the spread of heterochromatin. To investigate the enrichment of factors we calculated the active marker H3ac and the commonly suppressing marker H3K27me3 of the TAD boundaries. Indeed, we observed these elements at the boundary regions, which revealed that H3K27me3 had a clear segregation at the boundary region; otherwise, the H3ac was enriched in the boundary region (Additional file 4: Table S8). Interestingly, we also examined several regulatory elements in TAD boundaries, and the results showed that IRF2, CTCF, RREB1, REST, ZNF263 and FOXA1 were highly enriched in the control group of venom gland. Taken together, these results suggested that at the TAD boundaries, previously identified transcription factors and histone modifications that might potentially play an essential role in this process.

### Chromatin loops with histone markers are associated with highly expressed genes

**Fig. 4:**
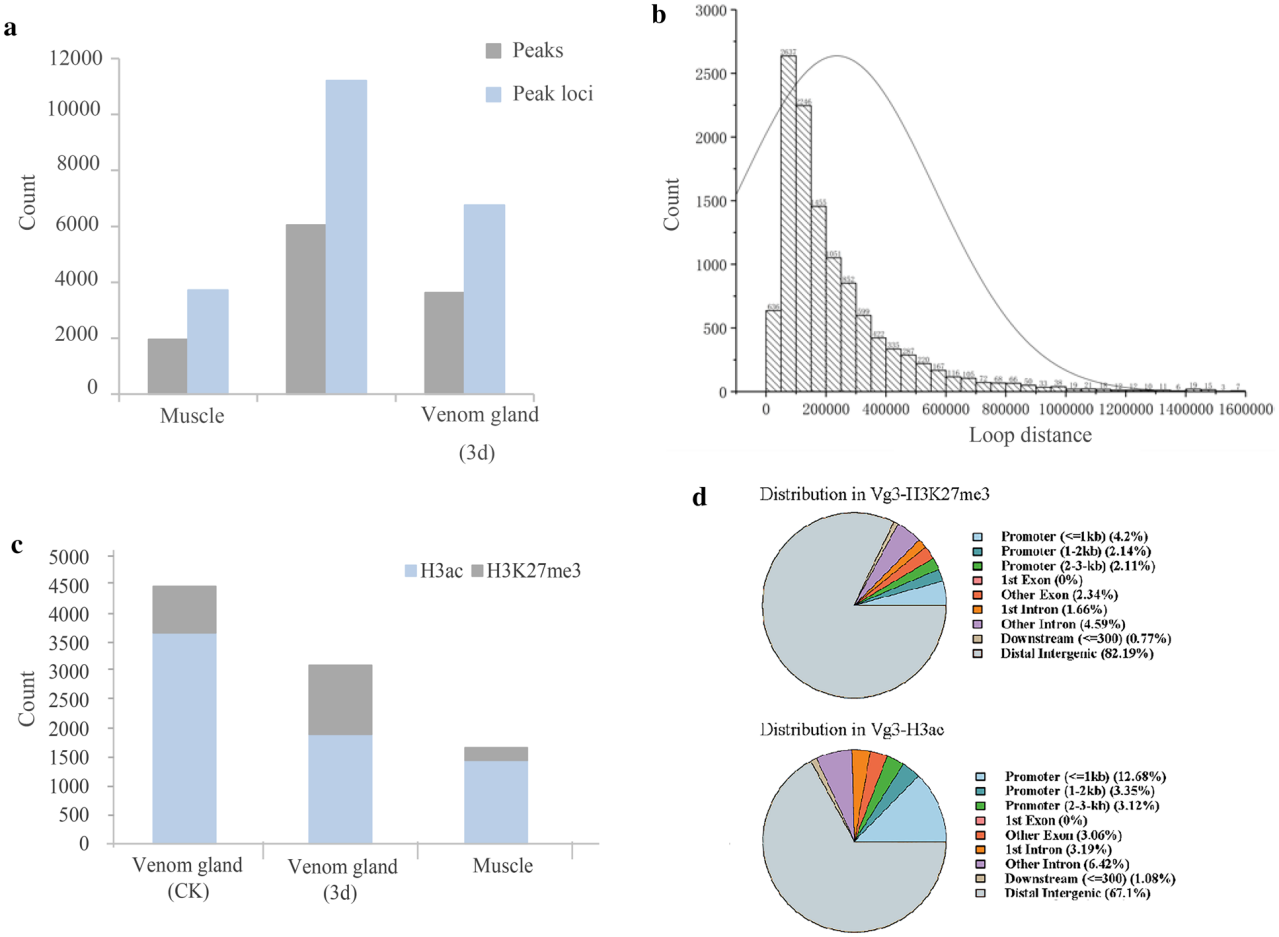
Chromatin loops with histone modifications in the time- and tissue-dependent groups are associated with highly expressed genes. **a** Histogram shows loop peaks and peak loci in gray and blue among muscle, the control and 3d group of venom gland, respectively. **b** The length distribution of loops. **c** The percentage of H3K27me3 (gray) and H3ac (blue) histone markers in muscle, the control group and the 3d group of venom gland located at euchromatic loops. **d** Distribution of histone markers H3K27me3 and H3ac Peak

Chromatin loops refer to the chromatin structures that bring the distant regulatory elements together. Chromatin loops can bring transcriptional enhancers and other transcription units together, in order to promote the recruitment of RNA PolII. According to the contacts on Hi-C interaction maps, we testified the chromatin loops at the 5 kb,10 kb and 25 kb resolution to ensured that most of the loops could be identified. Then, we measured the chromatin loop in *B. multicinctus* based on filtered Hi-C reads from our libraries. We detected 1951 loop peaks associated with 3711 distinct peak loci in muscle, 6083 loops associated with 11,213 distinct peak loci and 3623 loops coupled with 6750 distinct peak loci in the control and 3d group of venom gland, respectively (Fig. 4a). The vast majority of peaks (98.2%) identified loops between loci that are < 1 Mb apart. In A and B compartments, there were identified 6480 and 5171 chromatin loops were identified, respectively, which were mostly 50–100 kb in length. (Fig. 4b, Additional file 5: Table S9). Among those loops in A compartment, where H3ac was highly enriched as predicted, the formation of loops was related to functional gene annotations. In addition, euchromatic loops (loops located in the A compartment) were significantly enriched with the repressive marker H3K27me3 in 3d group of venom glands compared with the control group of venom gland and muscle, indicating that the formation of loops was determined by epigenetic markers (Fig. 4c, Additional file 5: Table S10). Accordingly, euchromatic loops were enriched with functional gene annotations, which can be reflected in high levels of promoter regions (Fig. 4d).

To explore the correlation between chromatin loops and the gene expression, following the gene expression level in *B. multicinctus*, we classified genome-wide genes by the same categorization method at the expression level in *Arabidopsis thalian*a [33]. The ratio of gene pairs by chromatin loops at the high expression level (FPKM > 20) was expressed much higher than that at the silence level. We used GO and KEGG pathway enrichment analysis to determine which gene functions and metabolic pathway loop-coupled genes were enriched. We determined that most genes enriched in loop regions encoded proteins with functions in RNA-related activities (positive regulation of angiogenesis, positive regulation of proteasomal ubiquitin—dependent protein catabolic process and negative regulation of intrinsic apoptotic signaling pathway) and essential metabolic pathways (carbon metabolism, zinc finger protein DZIP1, and biosynthesis of amino acids) in the 3d group of venom gland. For the control group of venom gland, Golgi membrane, protein transport, endoplasmic reticulum to Golgi vesicle—mediated transport and adaptive immune response were the main terms. In the muscle group, loop-coupled genes were more enriched in muscle and skeletal tissue development (actin cytoskeleton organization, filamentous actin, muscle alpha—actinin binding) and the MADS-box transcription enhancer factor pathway. Motifs enriched in loop peak loci included several regulatory elements, such as RREB1, IRF1, EWSR1-FLT1, etc.

We also detected histone modifications, including the active marker H3ac and the commonly suppressing marker H3K27me3. In total, 24,565/27,744/26,923 H3ac peaks and 24,051/8,684/5,162 H3K27me3 peaks were identified in 3d group of venom gland/the control group of venom gland/muscle ChIP-seq datasets. For venom gland, the lengths of peaks were approximately around 2,000 bp on average for H3ac and 500 bp for H3K27me3. For each marker, more than 10% of peaks were located in the gene promoter regions. Consistent with previous studies in other species [49–52], we found that the histone modification marks (H3K27me3 and H3ac) were mainly located around the TSS of active genes. Then, we calculated the number of H3ac/H3K27me3 at 1 kb region before transcriptional start sites (TSS), and 7.58%/12.46% in the muscle group and 18.97%/13.48% in the venom gland group were detected, respectively (Additional file 10: Figs. S7, S8). After the exploration of histone modifications, which are strongly associated with the expression of toxin-coding genes, we identified the biological processes by using GO enrichment. In the venom gland, H3ac was enriched in gene families such as protein folding (GO:0006457), intracellular protein transport (GO:0006886), chromatin silencing (GO:0006342), and H3K27me3 was enriched in mRNA splicing (GO:0000398), translation (GO:0006412), and rRNA processing (GO:0006364), following chromatin organization features.

### Distinct chromatin features are correlated with PLA2 family in *B. multicinctus*

**Fig. 5:**
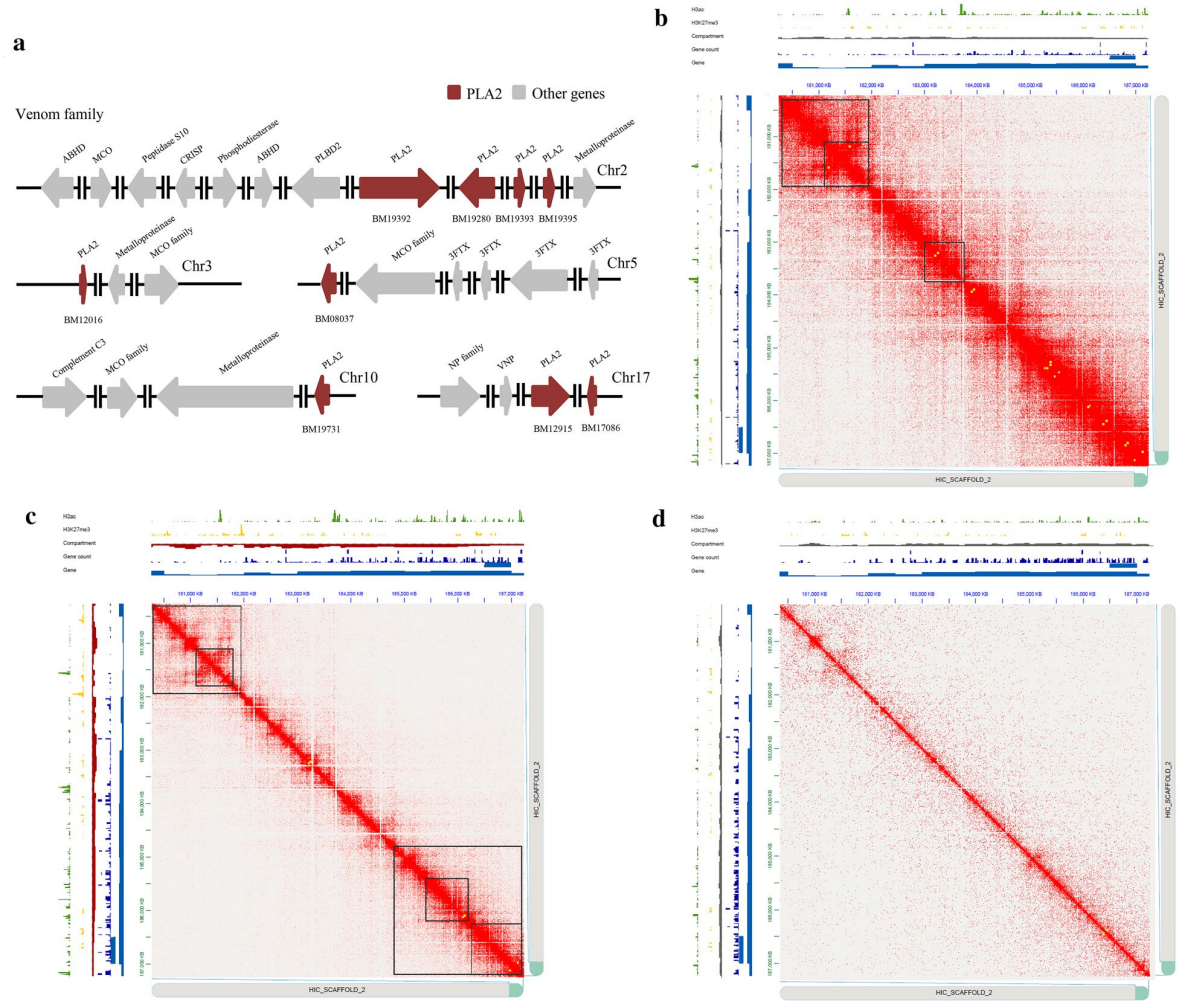
The expression and other chromosomal features of the PLA2 gene family in *Bungarus multicinctus*. **a** PLA2 gene family located on Chr2, Chr3, Chr5, Chr10 and Chr17. Red arrows, PLA2 gene family. Gray arrows, other venom gene families. A 7,000 kb region of Chr2 in *B. multicinctus* indicated the correlation of chromatin-related features. The chromatin features contain 5 classifications: active histone modifications, green; repressive histone modifications, yellow; the distribution of compartment, gray and red; gene expression, light blue; genomic location, blue in the control group of venom gland (**b**), the 3d group of venom gland (**c**) and muscle (**d**). The image was produced by Juicebox

The PLA2 family in *B. multicinctus*, it was presumed to exert the presynaptic or beta-neurotoxicity in elapids. In earlier work we identified 9 PLA2-encoding genes distributed on 5 chromosomes. Four genes (BM09280, BM19392, BM19393, BM19395) were tandemly arrayed on Chr2 as Group I PLA2(GI-PLA2s, also known as the elapid group). Two genes (BM17086 and BM12915) located on Chr17 were annotated as Group II PLA2s (GII-PLA2s, also known as the viperid group). The other three genes (BM08037, BM12016, BM19731) were annotated as GIB-PLA2s on Chr5, Chr3 and Chr10 (Fig. 5a).

To explore the correlation between epigenetics and the expression of toxin-coding genes, we detected distinct epigenetic features and examined the distributions of various histone modifications at different gene expression levels. After the foundation of 3D chromosome architecture at toxin-coding gene families in *B. multicinctus*, we began to establish the connection diagram among histone modifications, chromatin features, PLA2 gene family and gene expression in the control/3d group of venom gland and muscle. At a 7,000 kb region of Chr2 (180,500,000–187,500,000), we observed more intense interactions in the control group of the venom gland (Fig. 5b, c, d) and the interaction frequency between inter-chromosomes and intra-chromosomes was the same as that of the other 17 chromosomes. At the compartmental level, 16.9% of all compartments on Chr2 in muscle and the 3d group of venom gland compared with the control group of venom gland, changed from B compartment to A compartment. And we observed most compartments on the 7,000 kb region of Chr2 were closed (B compartment) on the 3d group of venom gland, where two Group I PLA2 genes were found (BM19393, BM19395). We detected 3/5 TADs and 25/18 loops in the control and 3d group of venom gland at 180,500,000–187,500,000. The active histone modification H3ac was highly enriched among the three groups, but we observed a more significant enrichment of the repressive histone marker H3K27me3 in the 3d group of venom gland. Interestingly, three genes (BM09280, BM19383, BM19385) located on the same loop were highly expressed, implicating the significance of epigenetic regulation in PLA2 expression.

## Discussion

Genomic DNA is not linear on chromosomes [53], and its 3D conformation plays an indispensable role in DNA replication, the regulation of gene transcription, chromatin concentration, and separation. The systematic epigenomic analysis provides a neoteric approach to obtaining a more comprehensive understanding of the genomic information. The proposed Hi-C technology and its large-scale application make it possible to reveal the interaction between different regulatory elements at the spatial level, and understand the mechanism and effect of chromatin conformation on the regulation of gene expression. Many epigenomic research projects have been implemented in humans and other model organisms to fully annotate functional genomic elements and to generate reference epigenome maps for various mammals, tissues, and cells [54]. *B. multicinctus* which has 18 chromosomes and a highly complex genomic architecture, still lacks of comprehensive epigenetic information. In this study, we have established the most complete 3D conformation and epigenomic map for *B. multicinctus* to date, which together provide a valuable basis for the research of chromatin hierarchy and epigenetic features on the support of snake genomes, the regulation of the dynamic expression of genes, and the limited context for snake venom regulation and evolution.

Comparison of time- and tissue-dependent Hi-C data revealed a significant difference between inter-chromosomal and intra-chromosomal contacts. One possibility for more interactions among MICs compared with MACs is that the randomization of individual contacts within the small chromosomes that tends to be much more intense in *B. multicinctus* genome, which causes two whole chromosomes to interact more than other two distant chromosomes. In Figures S2,3, and 4 and Table S2, no compensatory increase was observed among MACs, and between MACs and MICs, suggesting that it is not just a randomization of interactions and most of snake chromosomes have a similar likelihood of contacting each other. Through the analysis of gene clusters in the venom gland and muscle, there are more read-pairs in venom gland than it in muscle, and it could be implicating that different tissues of *B. multicinctus* actually harbor conserved chromosomal territories.

The delimitation of A compartments and B compartments are known to be ordinary chromatin features through the studies in various mammalian genomes. According to the relationship between increased gene expression in the transition of different times from one-type compartment to another and a high number of transitions on Chr2 which enriched with PLA2, we suggest that the potential mechanism for the phenomenon is probably due to the transcription differences of replenishment process of venom glands and toxin-coding genes, instead of the changes in chromosomal copy number among the time-dependent groups.

The genomic compartmental transition has proven to be correlated with gene expression. One assumption for compartmental and transcriptional changes that we found during different times in venom gland chromosomes, is that once a gene is activated or repressed in the process of detoxification of the venom glands, its position in the 3D nuclear space has already changed. The compartment also transforms to the opposite regions (A compartment to B compartment and vice versa). The above situation has been proven in previous microscopy studies [55]. As previously described, the conversion of the A/B compartment at different times is greater than that in various tissues within the conversional gene regions that are enriched with in activity and protein-folding related functions. Accordingly, this result provides a new evidence for the coordinated roles of venom gene regulation of chromatin organization and transcription factor activity.

According to the definitions of TADs, the separation of structural TADs is related to the switching boundaries of A/B compartment. In the past few years, it has been proven that the TADs are conserved and common in mammalian genomes since TADs were reported in humans and mice [56]. TADs in muscle can be fully found in the control group of venom gland which also shows the conservation of TADs in reptiles. Classical TAD-boundary regions are correlated with the transcription control in mammals [9]. Consistently, similar to the TADs in the control/3d group of venom gland and muscle in *B. multicinctus*, most of the TAD boundaries were the same. Compared with TADs, there were more DEGs in TAD boundaries where active/repressive histone modifications were enriched and many could be identified on Chr2. In combination with active modifications H3ac which were highly enriched in TAD boundaries, we suggest that heterogeneous epigenetic features could be related to the different distribution of genes and the gene expression levels in TAD boundaries and TAD-interior regions, indicating an intense connection between part of the topological chromatins and transcription [11, 13]. Surprisingly, there is a classical insulator (CTCF), three genes of PLA2 and toxin-coding transcription factors enriched in TAD boundaries, we hypothesize that CTCF is necessary for cycle regulation and structural development of tissues and that toxin-coding genes and transcription factors are involved in the synthesis and regulation of venom.

At a sufficient sequencing depth, finer chromatin loops can be detected [6, 15]. The active modification H3ac is enriched in A compartment among venom glands and muscle, but the highly expressed H3K27me3 is found in 3d group of venom gland in A compartment. Despite the hypothesis that various epigenetic marks influence the formation of chromatin loops, we also suggest that before the replenishment process, *B. multicinctus* may need to consume energy to hunt before envenomation which is reflected in the high enrichment of H3ac in the control group of venom gland. For the 3d group of venom gland, which was already prepared for subsequent hunting, H3K27me3 (Post-translational modification, PTM) was highly enriched to inhibit the recruitment of transcription initiation complexes, thereby inhibiting transcriptional regulation. It is evident that the chromatin looping in gene transcription is vital, and through this study, chromatin looping can be proven to be positively correlated with the expression of coupled genes. Through GO and KEGG analysis, the formation of chromatin looping with transcriptional regulation in RNA- and protein-related genetic functions can cause the underlying post-transcriptional regulation of downstream genes. In addition to being a chromatin structures, loops can also identify and prove gene interactions in reptiles, including interactions between enhancers, promoters, and insulator. This process is essential in the mechanism of producing toxin of *B. multicinctus,* for which we are still lack of the understanding of transcription patterns due to the lack of test groups within 0–3-day time intervals.

Compared with the findings of earlier studies of venom families among vipers [57, 58], *B. multicinctus* genome reveals the genomic location and context for snake PLA2 family genes and shows the PLA2 family, which is located on 5 chromosomes. To identify the roles of chromatin structure in toxin activity, we examined chromatin features and the expression level of the PLA2 family among the control/3d group of venom gland and muscle. Through Hi-C analysis, we found the evidence for tight regulation of chromatin in and around venom gene clusters. Moreover, on Chr2, which was enriched with the PLA2 family, toxin-coding genes occupied the venom-specific TADs, and 3 of toxin-coding genes were located on the same loop. In general, our results prove the coordinated roles of chromatin organization and transcription factor activity during the process of venom gene regulation.

## Conclusions

Overall, in this study, we plotted the chromatin architecture of *B. multicinctus* at different chromosome scales, from genome-wide chromosomal interactions to compartmentalization and the formation of TADs and loops. It is important to understanding how the epigenome regulates the formation of venom glands and muscle during development. Future studies that combine high-resolution analysis of chromatin structure with genetic experiments during the replenishment process can powerfully accelerate the understanding of the connection between venom gene regulation in vipers and hierarchical 3D genomes and the development of effective snake venom antiserum.

## Supplementary Information

**Additional file 1: Table S1**. Gene expression of venom glands relative to different times and various tissues.

**Additional file 2: Table S2**. Statistical results after aligned of hic data.

**Additional file 3: Table S3–S4**. The relationship between gene functions and specific expressed genes in venom gland

**Additional file 4: Table S5–S8**. A table listing the features of TADs and TAD boundaries between time- and tissue-dependent group.

**Additional file 5: Table S9–S10**. Chromatin loops in A/B compartments are related to histone modifications.

**Additional file 6: Figure S1**. Analysis of gene expression of different time and tissues. A. Scatter plot showing gene expression between the control group and 3d group in venom gland. The axes represent normalized RNA-seq log2FoldChange. Red and black dots denote genes whose expression changed significantly and grey dots denote genes whose expression was unchanged. B. The PCA analysis of 6 tissues in B. multicinctus.

**Additional file 7: Figure S2–S4**. Chromosomal interactions among control/ 3d group of venom gland and muscle. A. Comparison of intra-chromosomal interactions between MACs and MICs. B. Comparison of inter-chromosomal interactions between MICs and MICs-MACs. C. Comparison of inter-chromosomal interactions between MACs and MICs-MACs. D. Comparison of inter-chromosomal interactions between MACs and MICs.

**Additional file 8: Figure S5**. Compartmental conversion among chromosomes. A. The frequency of compartmental transition between 18 chromosomes in the control group of venom gland compared with the 3d group of venom gland. B. The frequency of compartmental transition between 18 chromosomes in muscle compared with the 3d group of venom gland.

**Additional file 9: Figure S6**. Relationship between histone proteins and genes. A. The relationship between transition of A/B Compartment and histone modifications. B. The Pearson correlation coefficients of H3ac and genes. C. The Pearson correlation coefficients of H3K27me3 and genes.

**Additional file 10**: **Figure S7–S8**. Heatmap and distribution of histone marks around TSS.

## Data Availability

Hi-C and ChIP-seq data are available from the National Center for Biotechnology Information Short Read Archive (SRA) under accession: PRJNA682532. Illumina RNA-seq data are available at NCBI under BioProject number: PRJNA606820.

## Abbreviations

3C: Chromosome conformation capture
Hi-C: High-througnput chromosome conformation capture
TAD: Topologically associated domain
CTCF: 11-Zinc finger protein or CCCTC-binding factors
LFC: Log2 fold change
Padj: P. adjust
ICE: Iterative Correction and Eigenvector Decomposition
PCA: Principal Components Analysis
MACs: Macro-chromosomes
MICs: Micro-chromosomes
PLA2: Phospholipases A2
IRF2: Interferon Regulatory Factor 2
RREB1: Ras Responsive Element Binding Protein 1
REST: RE1 Silencing Transcription Factor
ZNF263: Zinc Finger Protein 263;
FOXA1: Forkhead Box A1
EWSR1: EWS RNA binding protein 1
FLT1: Fms Related Receptor Tyrosine Kinase 1
PTM: Post-translational modification
FPKM: Fragments Per Kilobase of exon model per Million mapped fragments
GO: Gene Ontology
KEGG: Kyoto Encyclopedia of Genes and Genomes
ChIP-seq: Chromatin immunoprecipitation sequencingTSS: transcription start site
