# Supplementary material for: Hierarchical chromatin features reveal the toxin production in Bungarus multicinctus
Source: Chin Med. 2021 Sep 17;16:90. doi: 10.1186/s13020-021-00502-6 (PMC8447776; doi:10.1186/s13020-021-00502-6)
Supplement: Supplementary file 6 — Additional file 6: Figure S1. Analysis of gene expression of different time and tissues. A. Scatter plot showing gene expression between the control group and 3d group in venom gland. The axes represent normalized RNA-seq log2FoldChange. Red and black dots denote genes whose expression changed significantly and grey dots denote genes whose expression was unchanged. B. The PCA analysis of 6 tissues in B. multicinctus. [file 13020_2021_502_MOESM6_ESM.docx]

**Additional file 6: Figure S1.**


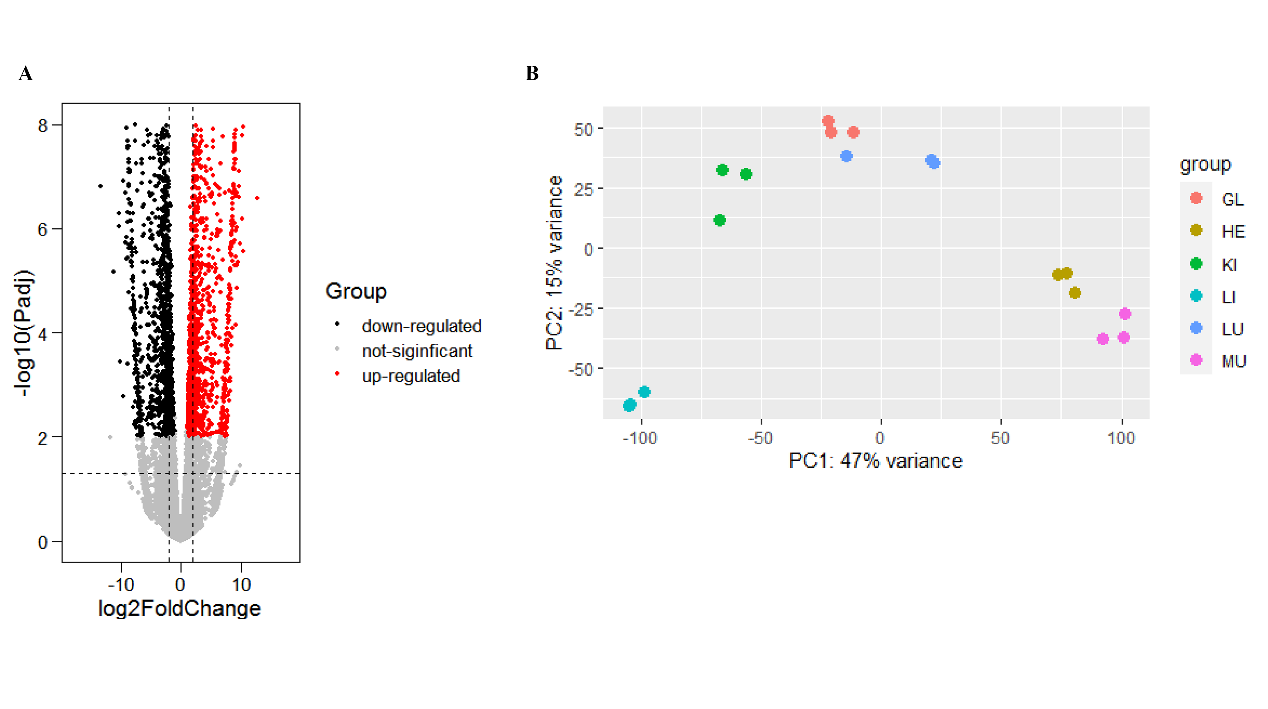
**Figure S1. Analysis of gene expression of different time and tissues.** a Scatter plot showing gene expression between the control group and 3d group in venom gland. The axes represent normalized RNA-seq log2FoldChange. Red and black dots denote genes whose expression changed significantly and grey dots denote genes whose expression was unchanged. B. The PCA analyses of 6 tissues in *B. multicinctus*.
