# Supplementary material for: Hierarchical chromatin features reveal the toxin production in Bungarus multicinctus
Source: Chin Med. 2021 Sep 17;16:90. doi: 10.1186/s13020-021-00502-6 (PMC8447776; doi:10.1186/s13020-021-00502-6)
Supplement: Supplementary file 7 — Additional file 7: Figure S2–S4. Chromosomal interactions among control/ 3d group of venom gland and muscle. A. Comparison of intra-chromosomal interactions between MACs and MICs. B. Comparison of inter-chromosomal interactions between MICs and MICs-MACs. C. Comparison of inter-chromosomal interactions between MACs and MICs-MACs. D. Comparison of inter-chromosomal interactions between MACs and MICs. [file 13020_2021_502_MOESM7_ESM.docx]

**Additional file 7: Figure S2-S4.**

**
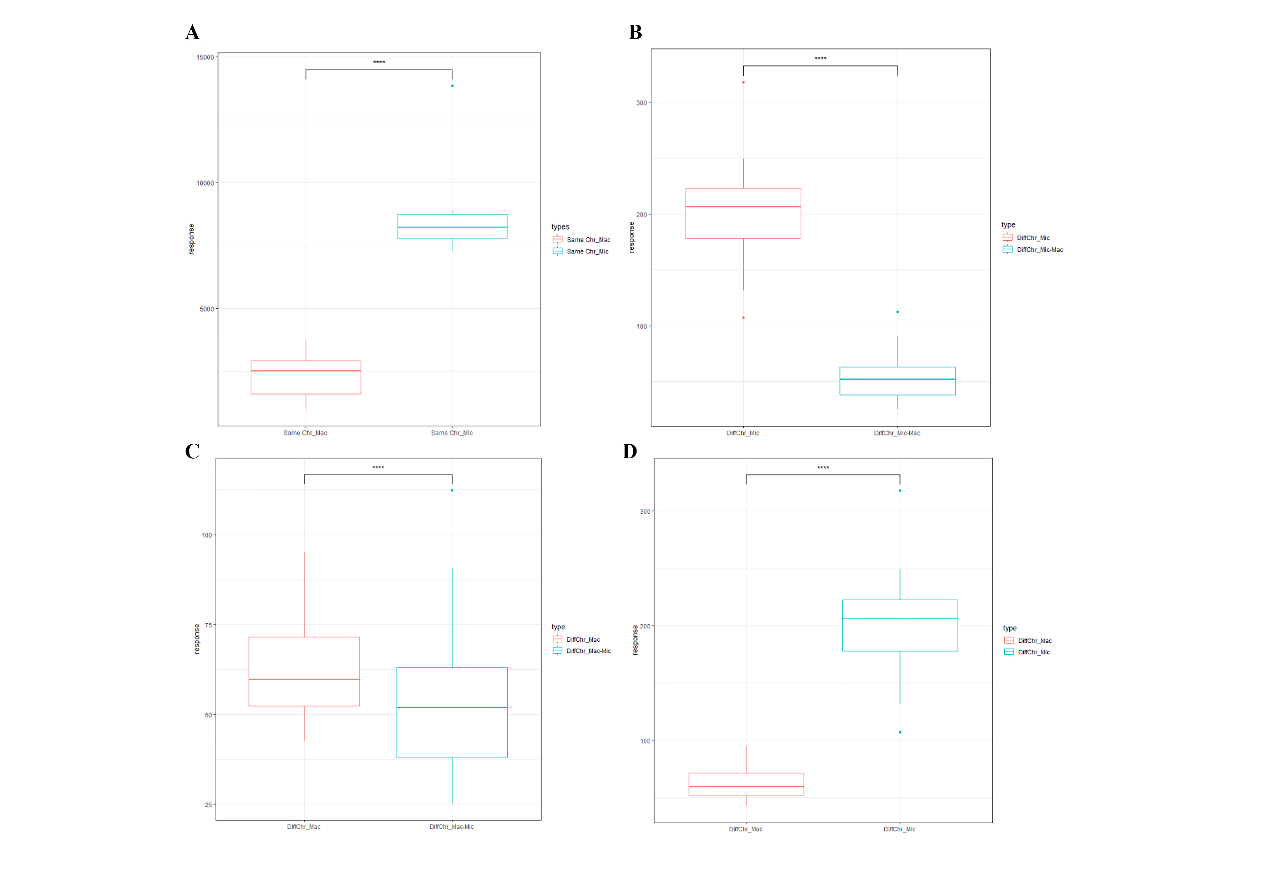
**

**Figure S2.** Chromosomal interactions in control group of venom gland. **A** Comparison of intrachromosomal interactions between MACs and MICs. **B** Comparison of inter-chromosomal interactions between MICs and MICs-MACs. **C** Comparison of inter-chromosomal interactions between MACs and MICs-MACs. **D** Comparison of inter-chromosomal interactions between MACs and MICs.

**
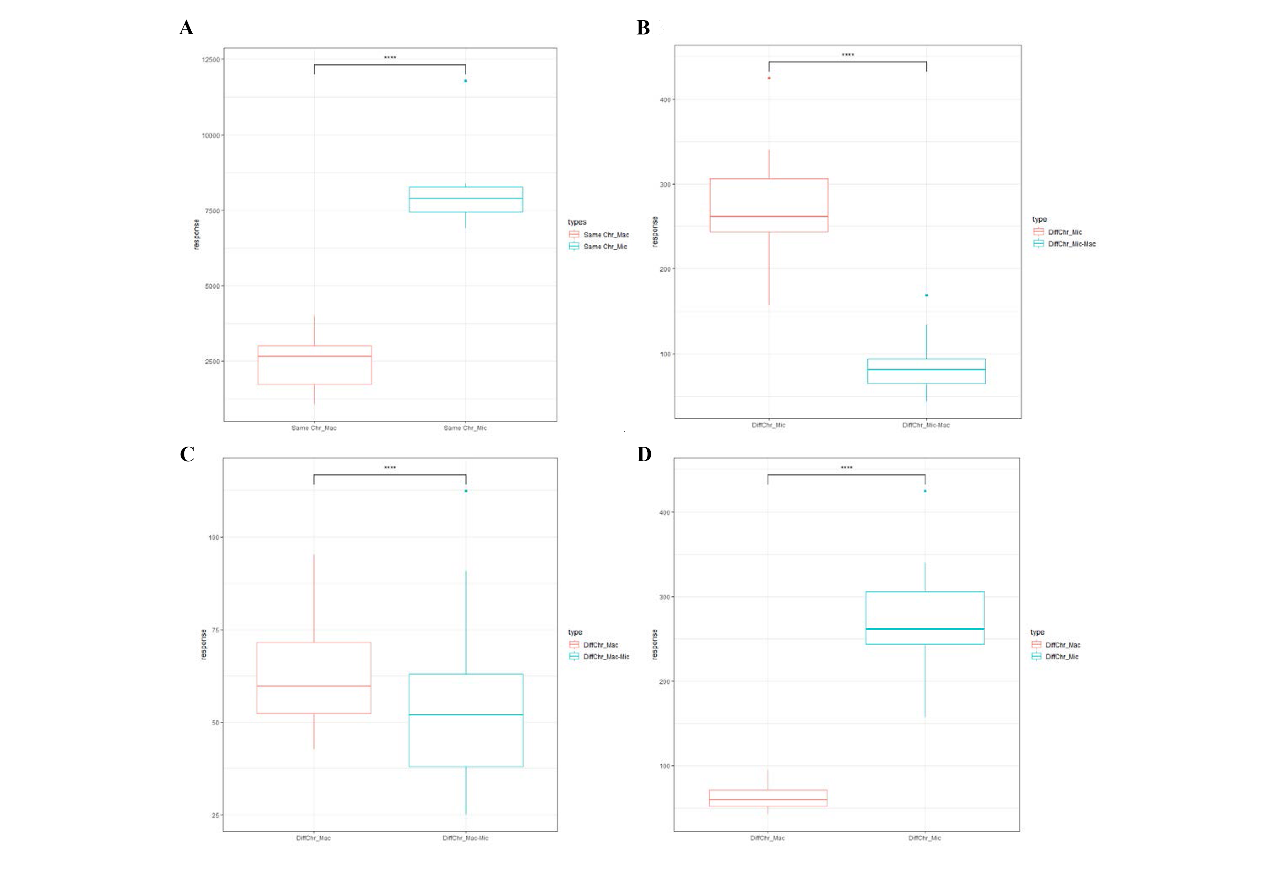
Figure S3.** Chromosomal interactions in 3d group of venom gland. **A** Comparison of intrachromosomal interactions between MACs and MICs. **B** Comparison of inter-chromosomal interactions between MICs and MICs-MACs. **C** Comparison of inter-chromosomal interactions between MACs and MICs-MACs. **D** Comparison of inter-chromosomal interactions between MACs and MICs.

**
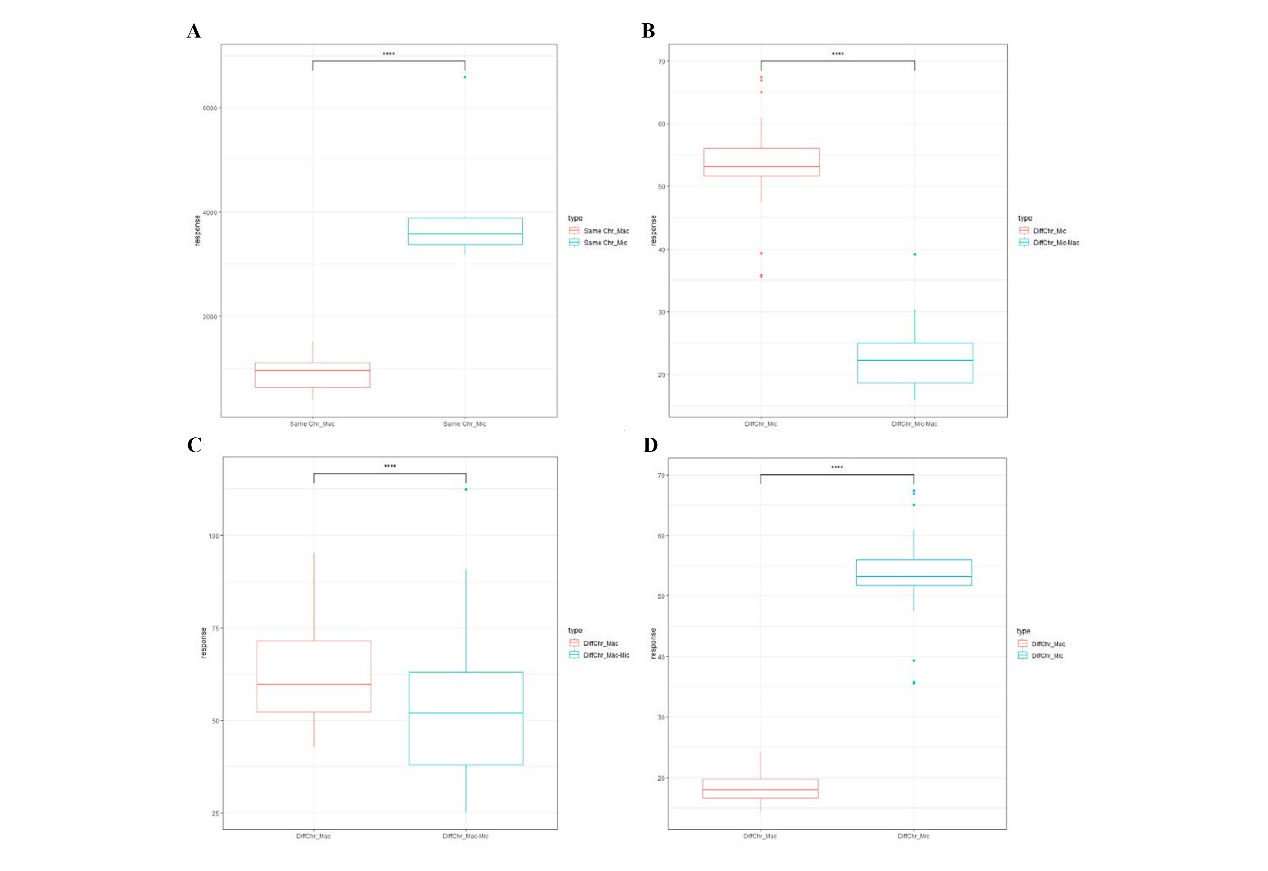
Figure S4.** Chromosomal interactions in muscle. A. Comparison of intrachromosomal interactions between MACs and MICs. B. Comparison of inter-chromosomal interactions between MICs and MICs-MACs. C. Comparison of inter-chromosomal interactions between MACs and MICs-MACs. D. Comparison of inter-chromosomal interactions between MACs and MICs.
