# Supplementary material for: Hierarchical chromatin features reveal the toxin production in Bungarus multicinctus
Source: Chin Med. 2021 Sep 17;16:90. doi: 10.1186/s13020-021-00502-6 (PMC8447776; doi:10.1186/s13020-021-00502-6)
Supplement: Supplementary file 8 — Additional file 8: Figure S5. Compartmental conversion among chromosomes. A. The frequency of compartmental transition between 18 chromosomes in the control group of venom gland compared with the 3d group of venom gland. B. The frequency of compartmental transition between 18 chromosomes in muscle compared with the 3d group of venom gland. [file 13020_2021_502_MOESM8_ESM.docx]

**Additional file 8: Figure S5.**


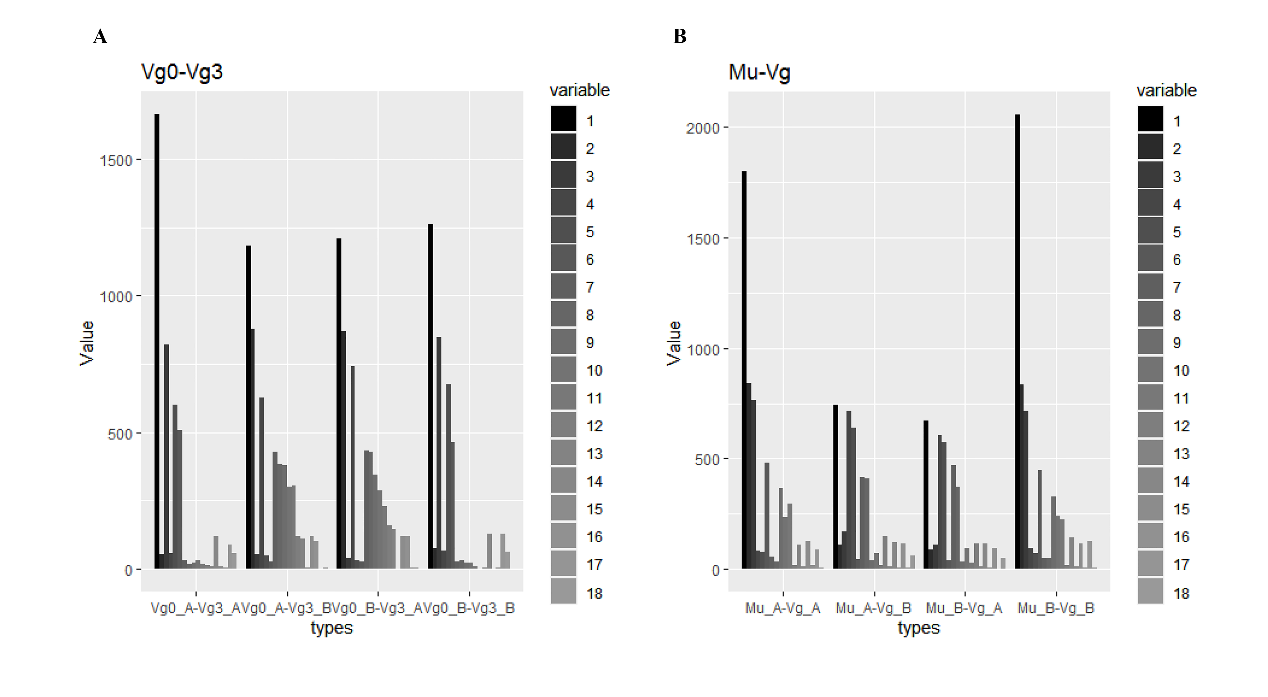


**Fig S5.** Compartmental conversion among chromosomes. A. The frequency of compartmental transition between 18 chromosomes in the control group of venom gland compared with the 3d group of venom gland. B. The frequency of compartmental transition between 18 chromosomes in muscle compared with the 3d group of venom gland.
