# Supplementary material for: Hierarchical chromatin features reveal the toxin production in Bungarus multicinctus
Source: Chin Med. 2021 Sep 17;16:90. doi: 10.1186/s13020-021-00502-6 (PMC8447776; doi:10.1186/s13020-021-00502-6)
Supplement: Supplementary file 9 — Additional file 9: Figure S6. Relationship between histone proteins and genes. A. The relationship between transition of A/B Compartment and histone modifications. B. The Pearson correlation coefficients of H3ac and genes. C. The Pearson correlation coefficients of H3K27me3 and genes. [file 13020_2021_502_MOESM9_ESM.docx]

**Additional file 9: Figure S6.**

**
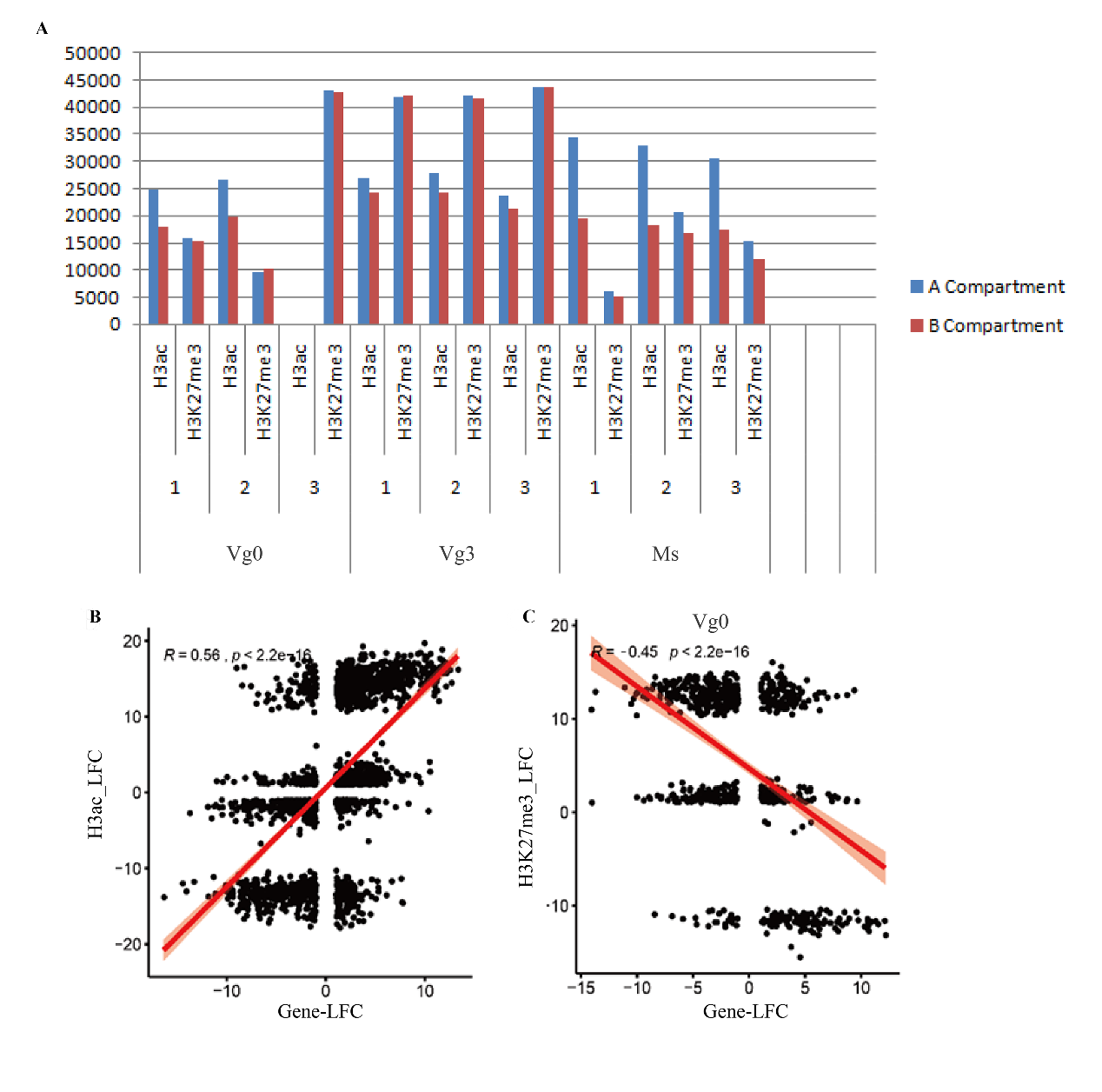
**

**Fig S6.** Relationship between histone proteins and genes. **A** The relationship between transition of A/B Compartment and histone modifications in the control group of venom, 3d group of venom gland and muscle, respectively. **B** The Pearson correlation coefficients of H3ac and genes. **C** The Pearson correlation coefficients of H3K27me3 and genes.
