# Supplementary material for: Hierarchical chromatin features reveal the toxin production in Bungarus multicinctus
Source: Chin Med. 2021 Sep 17;16:90. doi: 10.1186/s13020-021-00502-6 (PMC8447776; doi:10.1186/s13020-021-00502-6)
Supplement: Supplementary file 10 — Additional file 10: Figure S7–S8. Heatmap and distribution of histone marks around TSS. [file 13020_2021_502_MOESM10_ESM.docx]

**Additional file 10: Figure S7- S8.**


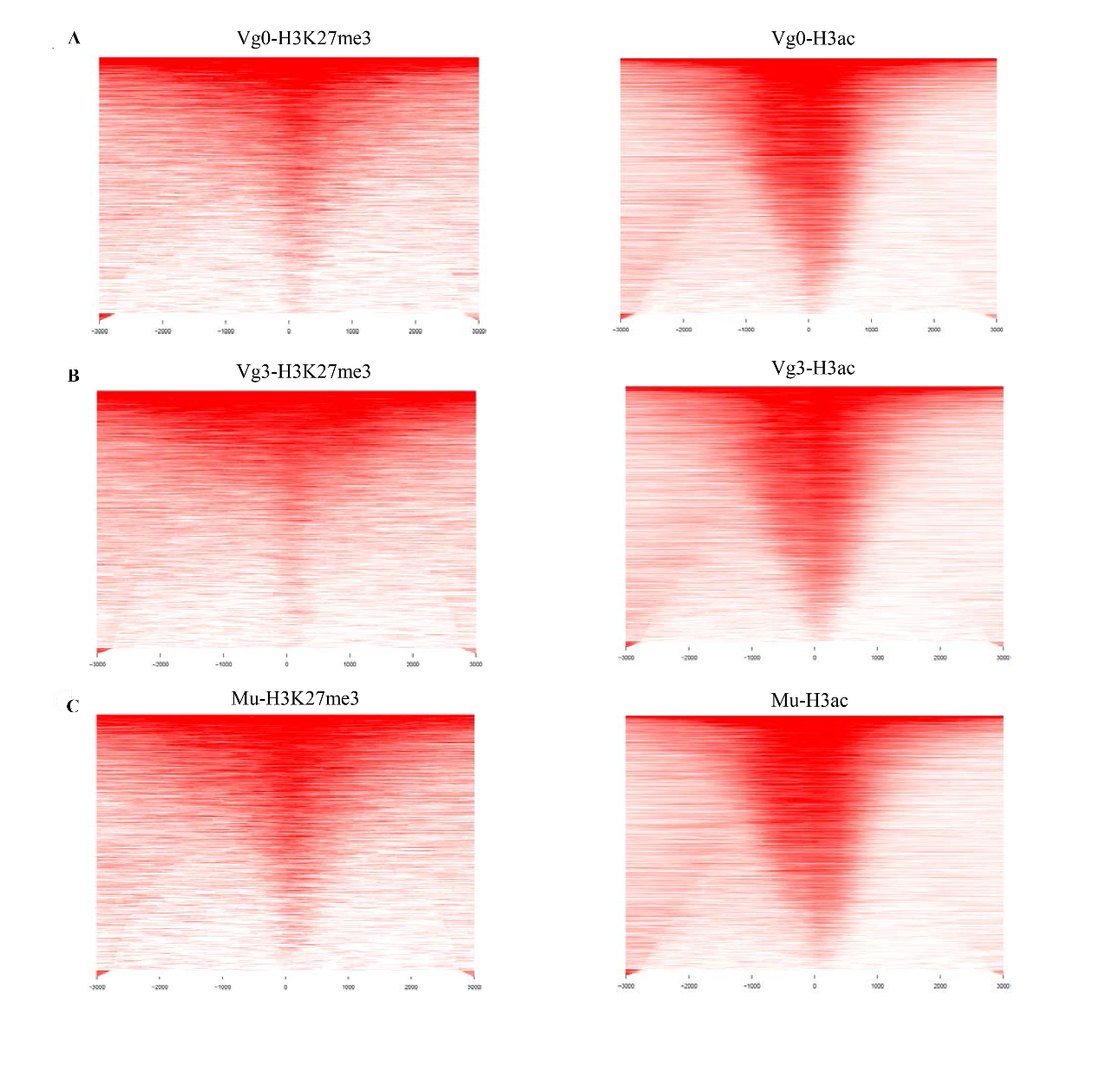
**Fig S7.** Heatmap of histone marks around TSS. **A** The heatmap of repressive/active histone markers in the control group of venom gland around TSS. **B** The heatmap of repressive/active histone markers in 3d group of venom gland around TSS. **C** The heatmap of repressive/active histone markers in muscle around TSS.


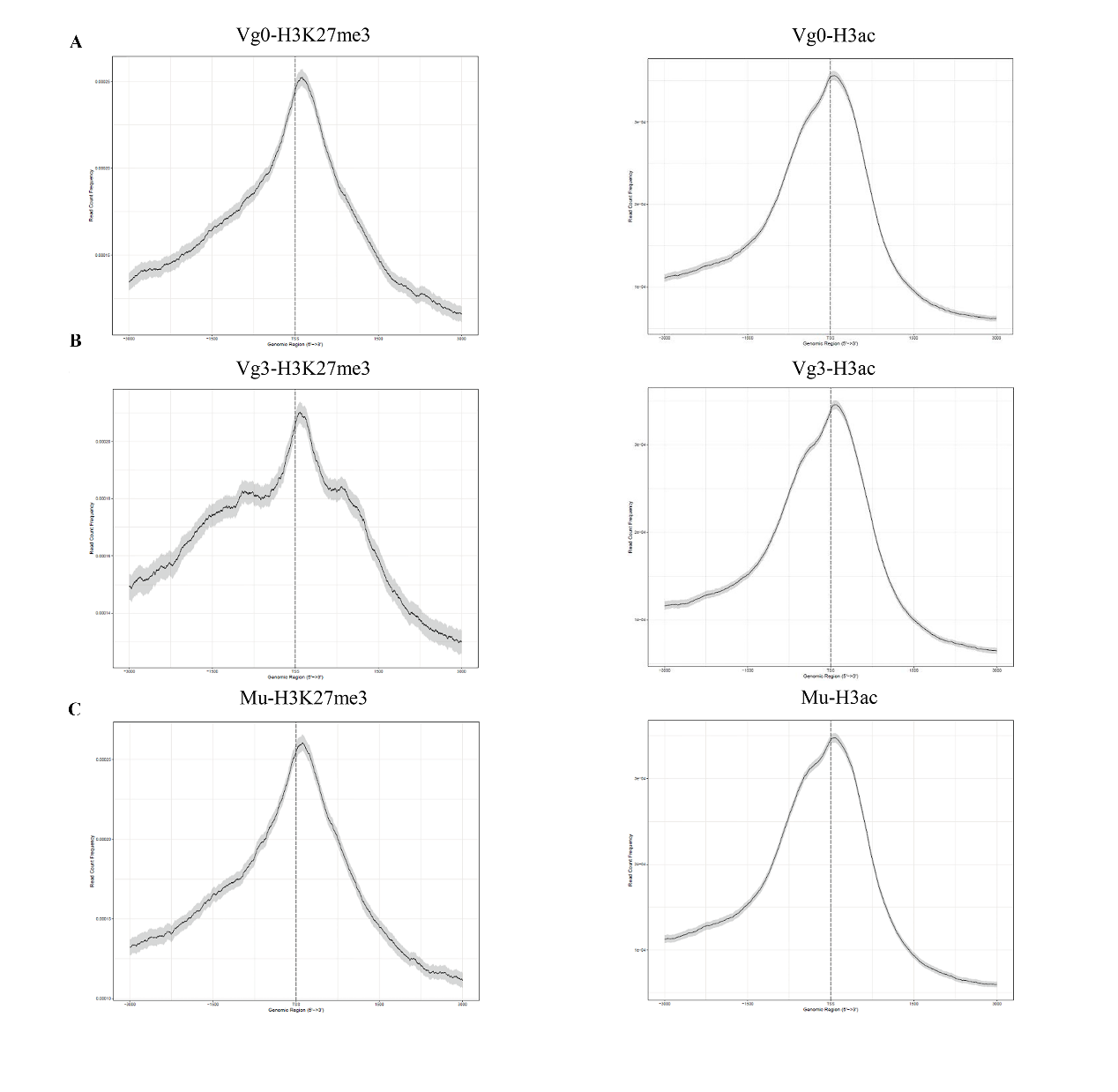
**Fig S8.** Distribution of histone marks. **A** The distribution of repressive/active histone markers in the control group of venom gland. **B** The distribution of repressive/active histone markers in 3d group of venom gland. **C** The distribution of repressive/active histone markers in muscle.
